# Intracellular bacteriolysis contributes to pathogenicity of *Staphylococcus aureus* by exacerbating AIM2-mediated inflammation and necroptosis

**DOI:** 10.1080/21505594.2022.2127209

**Published:** 2022-09-24

**Authors:** Shiyuan Feng, Yongjun Yang, Zhenzhen Liu, Wei Chen, Chongtao Du, Guiqiu Hu, Shuixing Yu, Peixuan Song, Jinfeng Miao

**Affiliations:** a Ministry of Education Joint International Research Laboratory of Animal Health and Food Safety, College of Veterinary Medicine, Nanjing Agricultural University, Nanjing, China; b Key Laboratory of Zoonosis Research, Ministry of Education, College of Veterinary Medicine, Jilin University, Changchun, China

**Keywords:** Bacteriolysis, AIM2, inflammasome, necroptosis, *staphylococcus aureus*, pathogenicity

## Abstract

*Staphylococcus aureus* can survive within phagocytes. Indeed, we confirm in this study that approximately 10% of population persists in macrophages during *S. aureus* infection, while the rest are eliminated due to bacteriolysis, which is of particular interest to us. Herein, we observe that the bacteriolysis is an early event accompanied by macrophage death during *S. aureus* infection. Furthermore, the cell death is significantly accelerated following increased intracellular bacteriolysis, indicating that intracellular bacteriolysis induces cell death. Subsequently, we establish that the cell death is not apoptosis or pyroptosis, but AIM2-mediated necroptosis, accompanied by AIM2 inflammasome activation. This finding challenges the classical model that the cell death that accompanies inflammasome activation is always pyroptosis. In addition, we observe that the apoptosis-associated genes are highly inhibited during *S. aureus* infection. Finally, we establish in vivo that increased bacteriolysis significantly enhances *S. aureus* pathogenicity by promoting its dissemination to kidney and leading to an inflammatory cytokine storm in AIM2-mediated manner. Collectively, our data demonstrate that bacteriolysis is detrimental when triggered in excess and its side effect is mediated by AIM2. Meanwhile, we propose a potential immune manipulation strategy by which *S. aureus* sacrifices the minority to trigger a limited necroptosis, thereby releasing signals from dead cells to inhibit apoptosis and other anti-inflammatory cascades of live cells, eventually surviving within host cells and establishing infection.

## Introduction

Macrophages are professional phagocytes populating and scrutinizing all tissues for signals disturbing homoeostasis, specifically, such as bacterial infection [1]. By sensing bacteria through surface or cytoplasmic pattern recognition receptors (PRRs), macrophages are able to categorize microbial invaders, initiate appropriate defence mechanisms in themselves and also alert other immune cells [1]. In order to realize these immune activities, macrophages employ the mechanisms of pathogen internalization for phagocytosis. This process involves the maturation of phagosome and the formation of phagolysosome that ultimately results in engulfed bacteria lysis. Following bacteriolysis, abundant bacterial contents are exposed to the host immune system, provoking a series of immune events, like inflammatory signalling cascades and effective antigen presentation. It indicates that bacteriolysis is not as simple as it defines.

Indeed, bacteriolysis by macrophages is a complicated process. First of all, it could be explained by that the phagosome biogenesis, accompanied by continuous fusion with trans-Golgi transport vesicles, endosomes, lysosomes, and autophagosomes, is highly regulated by a myriad of molecules [2]. Once internalized in phagosome, intracellular bacteria can killed by many ways, such as induction of toxic anti-microbial effector, acidification or metal accumulation in the phagolysosome. Additionally, the bacteriolysis accompanies multiple signalling cascades, such as inflammation and programed cell death pathway. However, its correlation with these signalling cascades has rarely been studied. To our best knowledge, inflammation is exquisitely modulated by various inflammasomes and helps host remove the injurious stimuli and initiate the healing process [3]. However, excessive inflammation will lead to tissue injury and disease [4]. The best characterized programmed cell death includes anti-inflammatory apoptosis [5], pro-inflammatory pyroptosis and necroptosis [6]. These cell death can protect the host against bacterial invaders, whereas their dysregulation has been implicated in a variety of autoimmune and auto-inflammatory conditions.

During coevolution, bacterial pathogens have evolved multiple strategies to circumvent host defence. Among these, *Staphylococcus aureus* is a remarkable representant, which employs a board array of virulence factors to challenge host immune system. For instance, *S. aureus* exploits the virulence of O-acetyltransferase A (*OatA*) to resist against the enzyme of lysozyme in phagocytes [7]. *S. aureus* evades phagocytosis by switching from planktonic growth to biofilm formation [8]. The staphylococcal proteins bind to the specific antibody and complement to interfere with opsonization in host [8]. These ability allows *S. aureus* to successfully survive phagosome and, ultimately, escape from macrophages. The escaped bacteria disseminates to seed other organs, leading to a large variety of diseases ranging from skin infections to life-threatening diseases, such as septicaemia, osteomyelitis and endocarditis [9]. During *S. aureus* infection, macrophages may play a vital role in *S. aureus* infection dynamics. Therefore, understanding the complex macrophage–pathogen interaction may provide promising new therapeutic targets.

To combat *S. aureus* in the clinical, antibiotics as the first discovered medicine have saved many lives. However, the emergence of antibiotic multi-resistant *S. aureus* strains has threatened the public health for the years [10]. To treat these more feared pathogens, new therapeutic approaches, such as antimicrobial peptides [11] and nanoparticles [12], have been developed. Of note, most of these therapeutic approaches, including antibiotics and antimicrobial peptides, are often linked to bacterial lysis, which indeed contributes to clearance of bacteria from host. However, based on our early findings, the host appears to be out of control when bacteriolysis is overwhelming. To explain this unusual situation, we magnify intracellular bacteriolysis of *S. aureus* by deleting lysozyme-resistance gene, *oatA*. By comparing the phenotype of wild-type and the mutant *S. aureus* strains in vitro and in vivo, we unveil the underlying mechanisms that may contribute the pathogenicity of *S. aureus*.

## Materials and methods

### Mice

C57BL/6J, AIM2-/-and Nlpr3-/- mice were purchased from the Jackson Laboratory (Bar Harbor ME, USA). Caspase-1/11^−/−^ and GSDMD^−/−^ mice were kindly provided by Prof. Feng Shao, National Institute of Biological Sciences, China [13]. MLKL^−/−^ mice were a gift from Dr. Jia-Huai Han, Xiamen University, China. All parental mice were backcrossed onto the C57BL/6J background for another eight generations. All animal studies were conducted according to the experimental practices and standards approved by the Animal Welfare and Research Ethics Committee at Jilin University (No. 20150601).

### Bacterial strains and growth conditions

*S. aureus* USA300-TCH1516 was used as parental strain. *S. aureus oatA* deletion mutant strain (Δ*oatA*) were generated by homologous arm exchange with plasmid pBT2 and *S. aureus oatA* complement strain (Δ::OatA) was obtained by transferring recombinant plasmid pLI50-*oatA* in Δ*oatA*. All the given strains were grown in brain-heart infusion (BHI) broth under 37°C, 200rpm.

### Murine infection

10 to 16-week-old female mice were injected subcutaneously in one flank with 3 × 10^6^ CFU of wild type *S. aureus* and simultaneously in the opposite flank with the same dose of Δ*oatA* for direct comparison. Lesion size, as assessed by the maximal length × width of the developing ulcers, was recorded daily. 10 to 16-week-old female mice were injected intravenously via tail with 1 × 10^8^, 0.5 × 10^8^, or 0.2 × 10^8^ CFU of *S. aureus*. Bacterial cultures (OD = 0.9) were washed, diluted, and resuspended in PBS.

### Cell infection

Bone marrow – derived macrophages (BMDMs) were sterilely isolated from 6- to 8-week-old female C57BL/6J mice and cultured in RPMI-1640 (Gibco) containing 25% L929 cell-conditioned medium for 7 days. *S. aureus* strains were grown to 0.9 of OD_600_, washed twice by PBS and adjusted to 1 of OD_600_. BMDMs cells were infected with *S. aureus* at multiplicities of infection (MOI = 50), centrifuged at 515 ×g for 4 min for synchronous infection, incubated at 37°C for 1 h, subsequently washed twice with PBS and added in fresh media containing 100 U/mL penicillin and 100 U/mL streptomycin.

### Intracellular bacterial survival assays

*S. aureus* strains were grown to 0.9 of OD_600_. Bacteria were washed twice by PBS and adjusted to 1 of OD_600_. Immediately, bacteria were added into 5 × 10^5^ BMDMs/500 μl/well in 24-well plate at MOI = 5, centrifuged at 515 × g for 4 min, and incubated at 37°C, 5% CO_2_ for 30 min. Wells were washed three times with PBS, and gentamicin (final concentration 300 μg/ml) was added in the fresh media to kill extracellular bacteria. After 1 h of incubation, media was replaced by fresh media containing 100 μg/ml gentamicin. At specified time points, BMDMs were washed three times with PBS, lysed in 0.01% Triton X-100, and CFU were calculated by plating on BHI-agar.

### Extracellular bacterial change assays

*S. aureus* bacteria were prepared as described in intracellular bacterial survival assays. Bacteria were added into 5 × 10^5^ BMDMs/500 μl/well in 24-well plate at MOI = 50, centrifuged at 515 × g for 4 min, and incubated at 37°C, 5% CO2. In parallel, to set control group, the same amount of bacteria were added into medium/500 μl/well in 24-well plate without BMDMs, centrifuged at 515 × g for 4 min, and incubated at 37°C, 5% CO2. After specified time of incubation, culture supernatants were collected and CFU were calculated by plating on BHI-agar. Fold change was obtained by comparing CFU of bacterial group and control group.

### Western blotting

BMDMs infected with bacteria were dissolved by lysis buffer containing 1% Triton X-100, 50 mM Tris-HCl (pH 7.4), 150 mM NaCl, 0.1 mM Na_3_VO_4_, 0.1 mM NaF, 1 mM PMSF, 5 mg/mL Aprotinin, 5 mg/mL Leupeptin, and 5 mg/mL Pepstatin A. Lysate and supernatant was harvested and used for immunoblotting analysis. The lysate (30 mg) were subjected to 12% sodium dodecylsulfate polyacrylamide gel electrophoresis and transferred onto polyvinylidene fluoride membranes (Millipore, Billerica, MA). After blocking with 5% milk, the membrane was blotted with antibodies against PARP (Cell Signalling), pMLKL (Abcam), RIPK3 (Abgent), Caspase-1 p20 (Adipogen), Cleaved caspase-3 (Cell Signalling), AIM2 (ebioscience), and β-Tubulin (Sungene Biotech, Tianjin, China).

### Cell death measurements

Cytotoxicity was evaluating by the measurement of lactate dehydrogenase (LDH) leakage from damage or destroyed cells (CytoTox 96 Non-Radioactive Cytotoxicity Assay Kit, Promega) and by propidium iodide (PI) incorporation assay.

### Statistical analysis

All values are expressed as means ±SEM. The statistical comparisons were performed using a one-way ANOVA with Dunnett’s multiple comparison test among multiple groups with a single control by the Prism software (GraphPad, La Jolla, CA). *p* values less than 0.05 (*p < 0.05, **p < 0.01, ***p < 0.001) were considered to be statistically significant.

## Results

### Intracellular bacteriolysis of Staphylococcus aureus triggers macrophage death

**Figure 1.**
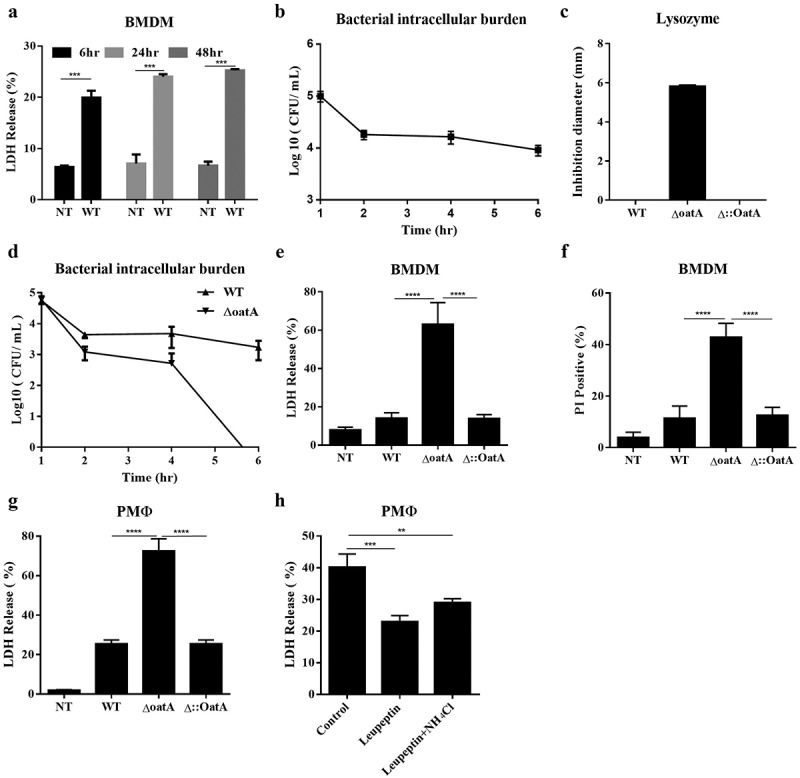
Increased intracellular bacteriolysis of *oatA*-deficient Staphylococcus triggers more cell death. (a) Lactate dehydrogenase (LDH) release assay of mouse bone marrow derived macrophages (BMDM) infected with wild-type *S. aureus* USA300 (WT) at MOI = 50 for 6 hr, 24 hr, or 48 hr, compared to BMDMs without infection (NT). (b) Intracellular bacteria survival in BMDM infected with WT. *T*-test, ***p < 0.001. (c) Lysozyme sensitivity of WT, *oatA* deficient mutant (Δ*oatA*) and complemented mutant (δ:oata) by agar diffusion assay. (d) Intracellular bacteria survival in BMDM infected with WT or δoata. (e) LDH release assay of BMDM infected with WT, δoata or δ::oata at MOI = 50 for 6 hr, compared to NT. (f) Propidium iodide (PI) incorporation assay of BMDM infected with WT, δoata or δ::oata at MOI = 50 for 6 hr, compared to NT. (g) LDH release assay of peritoneal macrophage (PMΦ) infected with WT, δoata or δ::oata at MOI = 50 for 6 hr, compared to NT. (h) LDH release assay of PMΦ pretreated with lysozyme inhibitors (Leupeptin, or Leupeptin plus NH_4_Cl) and subsequently infected with δoata. All the experiments were repeated at least three times with at least two technical replicates for each experiment. one-way ANOVA, *p < 0.05, **p < 0.01, ***p < 0.001, ****p < 0.0001.

To investigate whether intracellular bacteriolysis of *Staphylococcus aureus* triggers cell death in macrophages, we first stimulated mouse bone marrow-derived macrophages (BMDMs) with *S. aureus USA300 strain* for various times. Upon infection of macrophages, *S. aureus* activated a modest level of cell death at 6 h post infection determined by Lactate dehydrogenase (LDH) release assay. The death rate then remained constant until 48 h post infection (Figure 1a). It suggested that *S. aureus*-induced cell death was an early event and eventually *S. aureus* lived with macrophages in peace. Meanwhile, we observed that bacteriolysis occurred at the early stage of *S. aureus* infection determined by intracellular survival assay (Figure 1b). To further characterize bacteriolysis-induced cell death, we constructed a *S. aureus USA300 OatA* deficient mutant (Δ*oatA*), which was lysozyme-sensitive bacteria confirmed by extracellular growth inhibition (Figure 1c). *OatA* deficiency sharply decreased intracellular bacterial burden, indicating that bacteriolysis was increased (Figure 1d). Correspondingly, *OatA* deficiency magnified macrophage death determined by LDH release and Propidium iodide (PI) incorporation assays (Figure 1e,f). This effect was not specific in BMDM, but was also seen in peritoneal macrophage (Figure 1g). Moreover, the cell death induction could be inhibited by lysosome inhibitor leupeptin (Figure 1h). Collectively, these results indicate that increased intracellular bacteriolysis of *S. aureus* at the early stage of infection results in more cell death in the macrophage.

### Intracellular bacteriolysis triggers AIM2-mediated programmed necrosis

**Figure 2.**
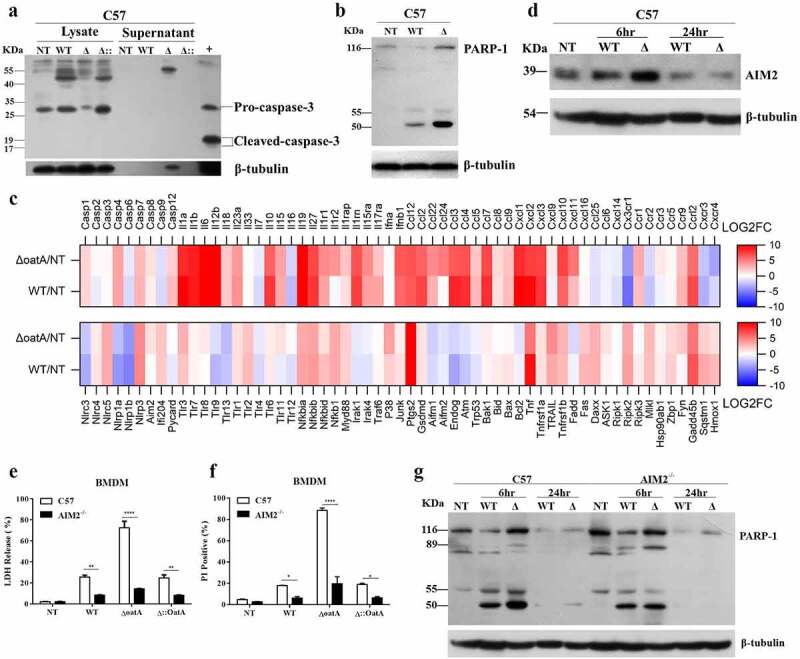
Intracellular bacteriolysis exacerbates AIM2-mediated programmed necrosis. (a) Western blot for cleaved-caspase-3 in BMDM infected with WT, δoata or δ:oata at MOI = 50 for 6 h, in contrast with BMDMs without infection (NT), + is the positive control. (b) Western blot for PARP-1 cleavage in BMDM infected with WT or δoata at MOI = 50 for 6 hr. Experiments were repeated three times. (c) Changes in the BMDM transcriptome infected with δoata or WT for 6 hr. Heatmap showed Log2 fold-changes of cell death and/or inflammatory response genes with a absolute Log2 (fold-change between δoata and WT stimulation group) > 1. (d) Western blot for AIM2 in BMDM infected with WT or δoata for 6 hr and 24 hr. LDH release (e) or PI incorporation (f) assays of C57 or AIM2-/- BMDM infected with WT, δoata or δ::oata at MOI = 50 for 6 hr. (g) Western blot for PARP-1 cleavage in C57 or AIM2-/- BMDM infected with WT or δoata at MOI = 50 for 6 hr and 24 hr. Each western blots was representative of several blots. The β-tubulin of (d) and (g) are identical because the representatives are from the same sample. Each cell death assay in each condition was repeated at least three times with at least two technical replicates. Two-way ANOVA, *p < 0.05, **p < 0.01, ***p < 0.001, ****p < 0.0001.

To characterize cell death, we first analysed the activation of caspase-3, the best recognized biochemical hallmark of apoptosis [14], on BMDMs infected with wild-type or Δ*oatA* mutant strain. No active caspase-3 was detected by western blotting, indicating that the cell death is not apoptosis (Figure 2a). Then, we analysed poly (ADP-ribose) polymerase (PARP-1) cleavage, which is decided by apoptosis and necrosis [15]. To execute apoptosis, PARP-1 is specifically proteolyzed by caspases to generate an 89 kDa C-terminal fragment and a 24 kDa *N*-terminal peptide [16]. Otherwise, PARP-1 is cleaved by lysosomal proteases to generate a major fragment of 50 kDa to perform necrosis [17]. Consistently, both wild-type and Δ*oatA* mutant strain stimulated PARP-1 necrotic fragment generation (Figure 2b). Moreover, the mutant strain, corresponding to increased intracellular bacteriolysis, induced higher levels of necrotic fragment generation compared to wild-type strain(Figure 2b), indicating that increased intracellular bacteriolysis-induced cell death is necrosis.

To determine all the genes of cell death and/or inflammatory response, transcriptome analysis was performed. First of all, we observed that apoptosis-associated genes, such as *Caspase-3*, *Aifm*, *Endog*, *Atm*, *Trp53*, *Bcl2, etc.*, were downregulated or no impact, while necrosis-associated genes, such as *Caspase-1/11, Gsdmd*, and *Mlkl* were upregulated (Figure 2c). It confirms that the macrophage death is non-apoptotic, but necrotic. Following analysis of all known PRRs available in the transcriptomic sequencing, *Aim2* gene was identified significantly upregulated upon Δ*oatA* mutant infection (Figure 2c). We further confirm this result by western blotting. Consistently, the mutant strain stimulated higher expression of AIM2 compared to wild-type strain (Figure 2d). To investigate the role of AIM2 on the cell death, we infected wild-type and *Aim2* gene-deficient (AIM2^−/−^) BMDMs with wild-type or Δ*oatA* mutant strain. Interestingly, AIM2 deficiency dramatically inhibited the cell death determined by LDH release (Figure 2e) and PI incorporation assays (Figure 2f). Additionally, in the absence of AIM2, the mutant-induced macrophage death is reduced by 5-fold while the wild-type strain-induced cell death is reduced by 3-fold, indicating the cell death is mediated by AIM2. Moreover, AIM2 deficiency abolished the production of PARP-1 necrotic fragments caused by increased intracellular bacteriolysis, as determined by western blotting (Figure 2g). These data indicate that bacteriolysis-induced necrosis is mediated by AIM2.

### Increased bacteriolysis exacerbates AIM2 inflammasome – mediated IL-1β release, but not pyroptosis

**Figure 3.**
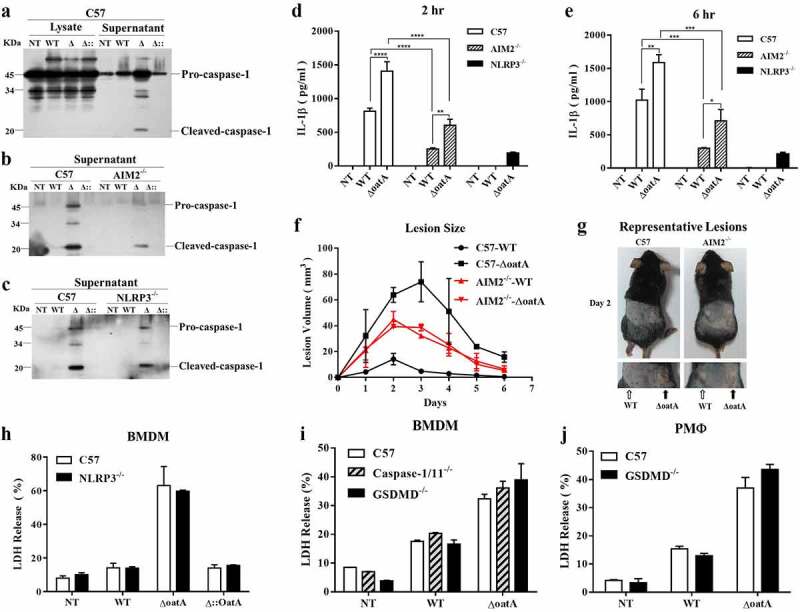
Intracellular bacteriolysis exacerbates AIM2 inflammasome activation, but not pyroptosis. (a) Western blot for cleaved-caspase-1 in lysate and supernatant of C57 BMDM infected with WT, δoata or δ:oata at MOI = 50 for 6 hr. (b) Western blot for cleaved-caspase-1 in supernatant of C57 or AIM2-/- BMDM infected with WT, δoata or δ::oata at MOI = 50 for 6 hr. (c) Western blot for cleaved-caspase-1 in supernatant of C57 or NLRP3-/- BMDM infected with WT, δoata or δ::oata at MOI = 50 for 6 hr. ELISA assay for IL-1β release of C57, AIM2^−/−^ or NLRP3^−/−^ BMDM pretreated with LPS for 4 hr and subsequently infected with WT or δoata at MOI = 50 for 2 hr (d) or 6 hr (e). (f) C57 or AIM2^−/−^ mice (n = 4) were injected subcutaneously in opposite flanks of notum with 3 × 10^6^ CFU of WT or δoata, and skin lesion size was measured at the indicated time. (g) a representative pair of lesions caused by WT or δoata after 2 days is depicted. (h) LDH release assay of C57 or NLRP3^−/−^ BMDM infected with WT, δoata or Δ::*OatA* at MOI = 50 for 6 h. (i) LDH release assay of C57, caspase-1/11^−/−^ or GSDMD^−/−^ BMDM infected with WT or δoata at MOI 50 for 6 hr. (j) LDH release assay of C57 or GSDMD^−/−^ PMΦ infected with WT, δoata or Δ::*OatA* at MOI 50 for 6 hr. Western blots were representative of several blots. ELISA assay was repeated twice with three technical replicates. Cell death assay was repeated three times with three technical replicates. Two-way ANOVA, *p < 0.05, **p < 0.01, ***p < 0.001, ****p < 0.0001.

According to the transcriptomic analysis, a number of genes involved in inflammation were identified as upregulated (Figure 2c). Therefore, we hypothesized that the cell death was related to inflammasome activation. To test such hypothesis, we first analysed caspase-1 cleavage, caused by inflammasome activation, upon infection of BMDMs with wild-type or Δ*oatA* mutant strain by western blotting. Cleaved caspase-1 was observed in the supernatant upon infection with the mutant (Figure 3a). In addition, given the fact that ASC speck is a direct evidence for inflammasome activation, we further analysed ASC oligomerization by western blotting. As shown in Supplementary Fig. S4, ASC dimer, corresponding to the ASC speck, was significantly increased upon infection with the mutant compared to wild-type strain. These data indicated that inflammasome activation was exacerbated by bacteriolysis. To characterize the resulting inflammasome activation, we analysed the phonotype of AIM2^−/−^ and NLRP3^−/−^ BMDMs infected with the wild-type or the mutant strain. We observed that AIM2 deficiency substantially decreased the production of the cleaved caspase-1 (Figure 3b), while NLRP3 deficiency had a small impact (Figure 3c). Given that IL-1β secretion depends upon the active caspase-1 processing following the activation of the inflammasome [18,19], we detected IL-1β secretion in wild-type, AIM2^−/−^ and NLRP3^−/−^ BMDMs pretreated with LPS and then infected with wild-type or the mutant strain. As expected, AIM2 deficiency sharply reduced IL-1β secretion and NLRP3 deficiency could not abolish IL-1β secretion upon infection of the mutant strain (Figure 3d,e). Collectively, these data indicate that bacteriolysis activates AIM2 inflammasome and leads to the resulting IL-1β secretion. Moreover, the time course of IL-1β secretion is compatible with the change of intracellular bacterial burden (Figure 1d), suggesting that AIM2 inflammasome activation is accompanied by intracellular bacteriolysis.

To in vivo verify the impact of bacteriolysis on inflammation, we constructed a skin infection model, which has been well established as an inflammation model in the literature [20]. Consistently, the mutant strain caused sizeable abscess lesions compared to wild-type strain in wild-type mice, and AIM2 deficiency significantly reduced the impact of the mutant strain (Figure 3f,g). Collectively, these data demonstrate that increased intracellular bacteriolysis exacerbates AIM2-mediated inflammation.

Given that Inflammasome activation is commonly accompanied by a specific programmed necrotic cell death, namely pyroptosis [13], we wondered whether the cell death described here is also in this case. As NLRP3 inflammasome was also activated by the mutant stimulation (Figure 3c), we firstly investigated whether NLRP3 is implicated in the cell death. However, NLRP3 deficiency had no impact on macrophage death determined by LDH release (Figure 3h). As the execution of pyroptosis depends on GSDMD that is cleaved and activated by caspase-1/11 [13], we then analysed the cytotoxicity in the absence of caspase-1/11 or GSDMD. Surprisingly, the deficiency of caspase-1/11 or GSDMD still had no impact macrophage death (Figure 3i,j). This effect was not specific in BMDM, but was also seen in peritoneal macrophage (Figure 3j). Collectively, these we demonstrate that increased bacteriolysis execrates AIM2 inflammasome – mediated IL-1β release, but not pyroptosis.

### Increased intracellular bacteriolysis exacerbates AIM2 mediated-necroptosis

**Figure 4.**
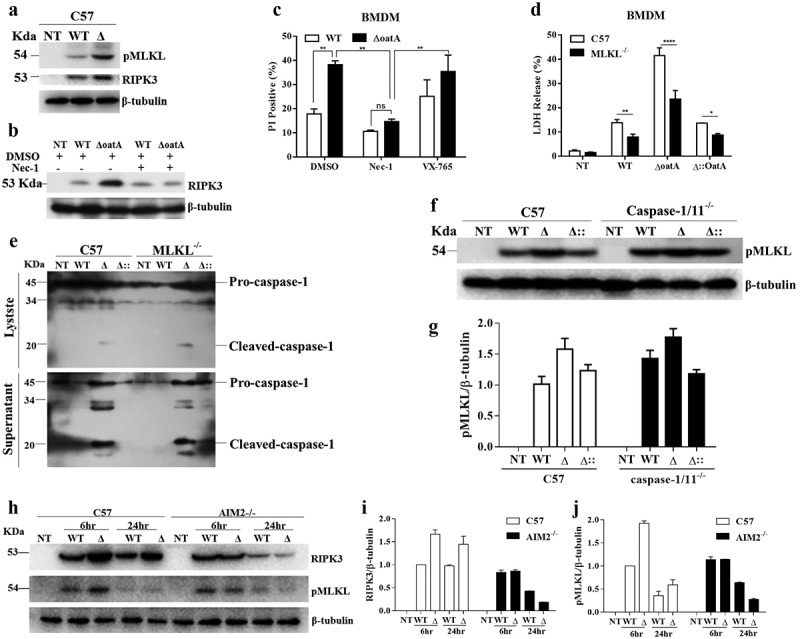
Intracellular bacteriolysis exacerbates AIM2-mediated necroptosis. (a) Western blot for RIPK3 and pMLKL in C57 BMDMs infected with WT or δoata at MOI 50 for 6 hr. (b) Western blot for RIPK3 in C57 BMDM pretreated with 50 μM nec-1 or DMSO for 1 hr and subsequently infected with WT or δoata at MOI 50 for 6 hr. (c) PI incorporation assays of C57 BMDM pretreated with 50 μM Nec-1, 20 μM VX-765 or DMSO and subsequently infected with WT or δoata at MOI 50 for 6 hr. (d) LDH assay of C57 or MLKL^−/−^ BMDM infected with WT or δoata at MOI 50 for 6 hr. (e) Western blot for cleaved-caspase-1 in C57 or MLKL^−/−^ BMDM infected with WT, δoata or δ:oata at MOI 50 for 6 hr. (f) Western blot for pMLKL in C57 or caspase-1/11^−/−^ BMDM infected with WT, δoata or δ::oata at MOI 50 for 6 hr. (g) Gray scanning analysis of Fig. 4 G. (h) Western blot for RIPK3 and pMLKL in C57 or AIM2-/- BMDM infected with WT or δoata at MOI 50 for 6 hr or 24 hr. (i) & (j) Gray scanning analysis of Fig. 4 H. Western blots were representative of several blots. Cell death assays were repeated three times with three technical replicates. Two-way ANOVA, *p < 0.05, **p < 0.01, ***p < 0.001, ****p < 0.0001.

As pyroptosis did not confirm to the mode of bacteriolysis-induced cell death, our attention was drawn to another best characterized programmed necrosis, called necroptosis. As established in the literature, necroptosis is executed via phosphorylated MLKL (pMLKL), which is mediated by RIPK [21]. Thus, we detected the production of pMLKL and RIPK3 in BMDMs infected with wild-type or the Δ*oatA* mutant strain by western blotting. Interestingly, both wild-type strain and the mutant stimulated pMLKL and RIPK3 expression compared to controls (Figure 4a), indicating that the cell death is necroptosis. Meanwhile, the mutant strain significantly induced higher levels of the expression of these two proteins (Figure 4a), indicating that increased bacteriolysis triggers necroptosis. This effect was not specific in C57 macrophage, but was also seen in BALB/c macrophage (Supplementary Fig. S5). To further confirm these results, we use necrostatin-1 (Nec-1), a necroptosis inhibitor, and MLKL deficient BMDMs to characterize the cell death. Consistently, RIPK3 production by the mutant strain was significantly inhibited by Nec-1 (Figure 4b). And also, the cell death was significantly inhibited by Nec-1 compared to VX-765, a pan-caspase inhibitor (Figure 4c), compatible with the results presented above. Furthermore, MLKL deficiency significantly decreased cell death rate (Figure 4d). Collectively, these data indicate that intracellular bacteriolysis-induced cell death is necroptosis.

To further characterize necroptosis, we first analysed the relationship between caspase-1 and MLKL. In the given experiments, we observed MLML deficiency did not impact the caspase-1 cleavage determined by western blotting (Figure 4e), and caspase-1/11 deficiency did not impact pMLKL production determined by western blotting (Figure 4f) and the corresponding grey scanning analysis (Figure 4g). It suggests that, unlike pyroptosis, the necroptosis is not dependent on caspases activation. Then, we analysed the role of AIM2 on the necroptosis. We detected RIPK3 and pMLKL production in wild-type and AIM2^−/−^ BMDMs infected with wild-type or the mutant strain by western blotting. Consistently, AIM2 deficiency abolished the increase of PIPK3 and pMLKL production induced by the mutant strain, determined by western blotting (Figure 4h) and grey scanning analysis (Figure 4i,j). It suggests that intracellular bacteriolysis-induced necroptosis is mediated by AIM2.

### Intracellular bacteriolysis contributes to S. aureus pathogenicity

**Figure 5.**
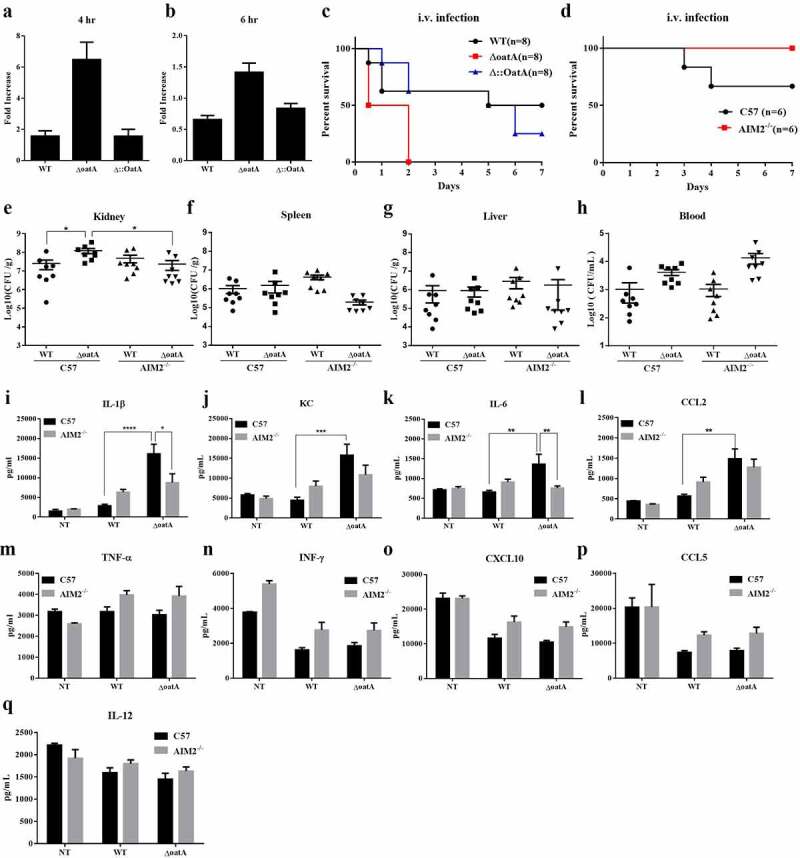
Increased intracellular bacteriolysis contributes to *S. aureus* pathogenicity. Extracellular bacterial change of BMDM infected with WT, ΔoatA or Δ::OatA at MOI=50 for 4 hr (a) or 6 hr (b) , compared with control group that had no cells and added bacteria. The experiments were repeated twice with three technical replicates. (c) Survival in mice following the tail intravenous (i.v.) injection of 1×108 colony-forming units (CFU) of WT, ΔoatA or Δ::OatA. (d) Survival in C57 and AIM2-/- mice following i.v. injection of 0.2×108 CFU of ΔoatA. Bacterial burden in kidney (e), spleen (f), liver (g) and blood (h) of C57 or AIM2-/- mice following i.v. of 0.5×108 CFU of WT or ΔoatA after 48 hr (n=8 per group, one-way ANOVA, *P<0.05). Pro-inflammatory mediators in kidney of C57 or AIM2-/- mice at 48 hr post i.v. infection of 0.5×108 CFU of WT or ΔoatA were examined by ELISA assay : (i) IL-1β, (j) KC, (k) IL-6, (l) CCL2, (m) TNF-α, (n) IFN-γ, (o) CXCL10, (p) CCL5, (q) IL-12. Two-way ANOVA, *P<0.05, **P<0.01, ***P<0.001, ****P<0.0001.

To assess the significance of intracellular bacteriolysis on *S. aureus* infection, we firstly analysed extracellular bacterial change of BMDMs infected with wild-type or mutant strains by comparing with control group that had no cells and added bacteria. In the given experiments, the mutant exhibited higher levels of extracellular bacterial change than the wild-type bacteria (Figure 5a,b), even though there was less difference between the mutant and the wild-type at 6 h post-infection due to extracellular bacterial growth (Figure 5a). It indicates that necroptotic cell death contributes to release of bacteria. Accordingly, we hypothesized that intracellular bacteriolysis might contribute to its dissemination during *S. aureus* infection. To test this hypothesis, we constructed a murine systemic infection model by intravenous (i.v.) injection. Consistently, the mutant strain dramatically increased mortality of wild-type mice compared to wild-type strain (Figure 5c), indicating that increased host cell death due to bacteriolysis is linked to the increased mortality. To properly perform subsequent analysis, we adjusted the infection dose to a modest level. Then, we compared the mortality of wild-type and AIM2^−/−^ mice upon i.v. infection with the mutant strain. Consistently, AIM2 deficiency can decrease mice death rate (Figure 5d). To further characterize the mice death, we used an appropriate dose of wild-type or mutant strain to infect wild-type and AIM2^−/−^ mice. At 2 days post infection, we analysed the bacterial burden in organs and only observed a significant difference in kidney (Figure 5e) and an unsure difference in blood stream (Figure 5h). In details, in wild-type mice, the mutant strain infection led to higher levels of bacterial burden in kidney and blood compared to wild-type strain infection (Figure 5e,h). This result is compatible with the extracellular bacterial change (Figure 5a,b), indicating that cell death contributes to *S. aureus* dissemination. In parallel, consistently, AIM2 deficiency could prevent the increase of kidney bacterial burden due to the mutant infection, indicating that the resulting bacterial dissemination is mediated by AIM2 (Figure 5e). To investigate whether inflammation is involved, we detected the release of cytokines and chemokines in kidney collected from the above experiments. Interestingly, the mutant strain consistently induced much higher levels of IL-1β, KC, IL-6 and CCL2 release in wild-type mice (Figure 5i–l), indicating that an inflammatory cytokine storm is induced by the mutant strain due to increased bacteriolysis. As expected, AIM2 deficiency could prevent the emergence of the cytokine storm (Figure 5i–l), indicating that the inflammatory cytokine storm is mediated by AIM2. Collectively, these results suggests that intracellular bacteriolysis contributes to pathogenicity of *S. aureus* by exacerbating AIM2-mediated inflammation and necroptosis.

## Discussion

*S. aureus* is commonly considered to be an extracellular pathogen, but also able to survive within host cells. To explain this survivability of *S. aureus*, we decided to start the study with the bacteriolysis that is the main cause of the loss of this pathogen in phagocytes.

In this study, we establish that the bacteriolysis is an early event accompanied by cell death and AIM2 and NLRP3 inflammasomes activation. It is not surprise that NLRP3 inflammasome is found activated by bacteriolysis. In line with this finding, Shimada T *et al*. have demonstrated that the particulate peptidoglycan via bacteriolysis activates NLRP3 inflammasome and therefore leads to IL-1β secretion during *S. aureus* infection [20]. In addition, they found increased bacteriolysis induces more IL-1β-dependent skin information. This is consistent with our findings in skin infection model (Figure 3f,g). However, we further unveil that AIM2 inflammasome also contributes to IL-1β secretion *in vitro* and in skin or systemic infection. Moreover, besides IL-1β, other key inflammatory mediators, including IL-6, KC and CCL2, were found controlled by AIM2 during systemic infection (Figure 5i–l). Interestingly, these results are consistent with the work of Hanamsagar, Richa *et al*. on central nervous system infection of *S. aureus* [22]. In their work, they also found IL-1β production is mediated throuth the NLRP3 and AIM2 inflammasome and the secretion of IL-6, KC and CCL2 is dependent on AIM2. Taken together, it indicates that AIM2 inflammasome plays a critical role on *S. aureus* infection and the host response to this pathogen may not be affected by the way it invades. Inflammasome activation is commonly accompanied by pyroptosis [13]. Unexpectedly, the resulting cell death in our study is identified as necroptosis, which is also considered as a pro-inflammatory cell death and has a closed relationship with inflammasome. Indeed, it has been reported that activated MLKL mediated NLRP3 inflammasome activation and IL-1β release [23]. In our case, the necroptotic activity of MLKL requires on cytosol AIM2 inflammasome, providing another evidence for the closed relationship between necroptosis and inflammasome.

Herein, it is interesting to mention the previous work of Peng et al. In their study, increased intracellular bacteriolysis of *Francisella tularensis* induced hyper-cytotoxicity in macrophages and triggered AIM2 inflammasome-dependent pyroptosis [24]. Oddly, similar beginnings lead to different endings. However, there are a few significant differences between two studies. First, most obviously, the bacterial specie is different: *Francisella tularensis* is gram-negative and *S. aureus* is gram-positive. Second, different genes are chosen to construct the mutants. Thus, macrophages may deal with bacteria differently. Third, most importantly, AIM2 expression is totally different. In their study, there is no difference in AIM2 transcript and protein between mutant-infected and wild-type-infected macrophages. On the contrary, we observe that mutant triggers much higher levels of AIM2 transcription and translation (Figure 2c,d). With those differences, we believe that there is a different signalling pathway underlying between different bacteria species. Indeed, the study of Camille Aubry et al. in *Listeria monocytogenes* provides a perfect evidence. In their study, *oatA* deletion strongly attenuated *Listeria* virulence in mice following intravenous inoculation [25], whereas *oatA* deletion drastically increased *S. aureus* virulence under the same condition (Figure 5c).

In addition, a few previous studies have investigated a seemingly similar process that phagocytosis of *S. aureus* induces programmed necrosis or lysis of host cells [26–30]. They established the survivability of *S. aureus* in human phagocytes, mainly in neutrophils, but did not understand the underlying mechanism as deep as we did. Interestingly, the lysis of human neutrophils fed *S. aureus* in these studies [27–30] requires RIPK3 but is independent of MLKL [29], while the macrophages necrosis presented in our study is dependent on MLKL. This difference can be explained by the different investigated cells. It has been well established that the stepwise succession of phagosomal maturation in macrophages is strikingly different from phagosome formation in neutrophils [31].

Regarding signals triggering the necroptosis, bacterial DNA is a perfect candidate as it can be sensed by the cytosolic DNA sensor AIM2. According to our extra data, we obverse that bacterial DNA or synthetic double-stranded DNA can induce AIM2-medicated macrophage death only under conditions of co-transfection with the cationic lipid DoTAP (Supplementary Fig. S1). More interestingly, the co-transfection with HLA and DoTAP also induced AIM2-mediated cell death, while only HLA stimulation had no impact (Supplementary Fig. S2). Why is AIM2 able to simultaneously mediate DNA and HLA induced-necroptosis? One possible explanation is that AIM2 can sense both DNA and HLA. Thanks to bacteriolysis, diverse PAMPs, including DNA and HLA, are released from bacteria, providing the possibility for AIM2 to access different signals. Otherwise, AIM2 might mediate necroptosis directly by sensing bacteriolysis machinery, such as phagosome or lysosome. Further studies will be necessary to establish this part of the underlying mechanism.

In addition to the signals released from bacteria, interestingly, known from our transcriptomic analysis, TRAIL-TRALR, cell death signal and receptor, are identified as upregulated (Figure 2c). Typically, TRAIL, known as tumour necrosis factor ligand superfamily member 10, binds to its receptor TRALR, leading to apoptosis [32]. Meanwhile, it has been revealed that TRAIL-stimulation, in the presence of caspase inhibitors, can be shifted to RIPK1/RIPK3/MLKL-dependent necroptosis [33]. Consistently, in our study apoptosis is strongly inhibited, as demonstrated by showing that numerous apoptosis-associated genes, such as *FADD*, *Aifm* and *Endog*, are transcriptionally downregulated and the key effector of apoptosis, caspase-3, is not activated. Therefore, TRAIL-TRALR signals in our case also undergoes necroptotic pathways, as a result, accelerating the death of the responding cells.

Finally, we draw conclusions from two perspectives. To host, bacteriolysis by macrophages is a direct bacterial killing. However, it is detrimental when triggered in excess. For the reason, increased bacteriolysis exacerbates tissue injury and even host death during *S. aureus* infection. The side effect of increased bacteriolysis involves excessive inflammation, which is mainly driven by AIM2-mediated inflammasome activation and necroptosis. It has been established that excessive or subnormal inflammation is a major driver of diseases [4]. Therefore, our findings suggest that AIM2 signalling pathway might be a therapeutic target to control unwelcome inflammation. To pathogen, bacteriolysis contributes to pathogenicity of *S. aureus*. As nicely review by Dominique Missiakas and Volker Winstel [34], *S. aureus* can manipulate apoptotic, necroptotic and pyroptotic cell death modes to establish infection in different environment where host defence arsenals may be triggered differently. Here, we provide another evidence for this notion. In our case, wild-type *S. aureus* allows a small portion of the bacteria to be lysed, which results in limited necroptosis and reduced inflammation so that the remaining bacteria can persist longer. Moreover, apoptotic signalling cascades are strongly inhibited during infection. Apoptosis is known to be important for clearing pathogens [35]. Collectively, we propose that *S. aureus* may utilize the lysed bacteria to trigger limited necroptosis, which may release signals from dead macrophages to inhibit apoptosis and other anti-inflammatory cascades of live cells, eventually surviving within host cells and establishing infection [36]. This may be to some extent supported by the dynamic change of *oatA* transcription levels during *S. aureus* infection (Supplementary Fig. S3).

## Supplementary Material

Supplemental Material

## Data Availability

Data sharing is not applicable to this article as no new data were created or analysed in this study
