# Supplementary figures and images for: Intracellular bacteriolysis contributes to pathogenicity of Staphylococcus aureus by exacerbating AIM2-mediated inflammation and necroptosis
Source: Virulence. 2022 Sep 24;13(1):1684–96. doi: 10.1080/21505594.2022.2127209 (PMC9519016; doi:10.1080/21505594.2022.2127209)

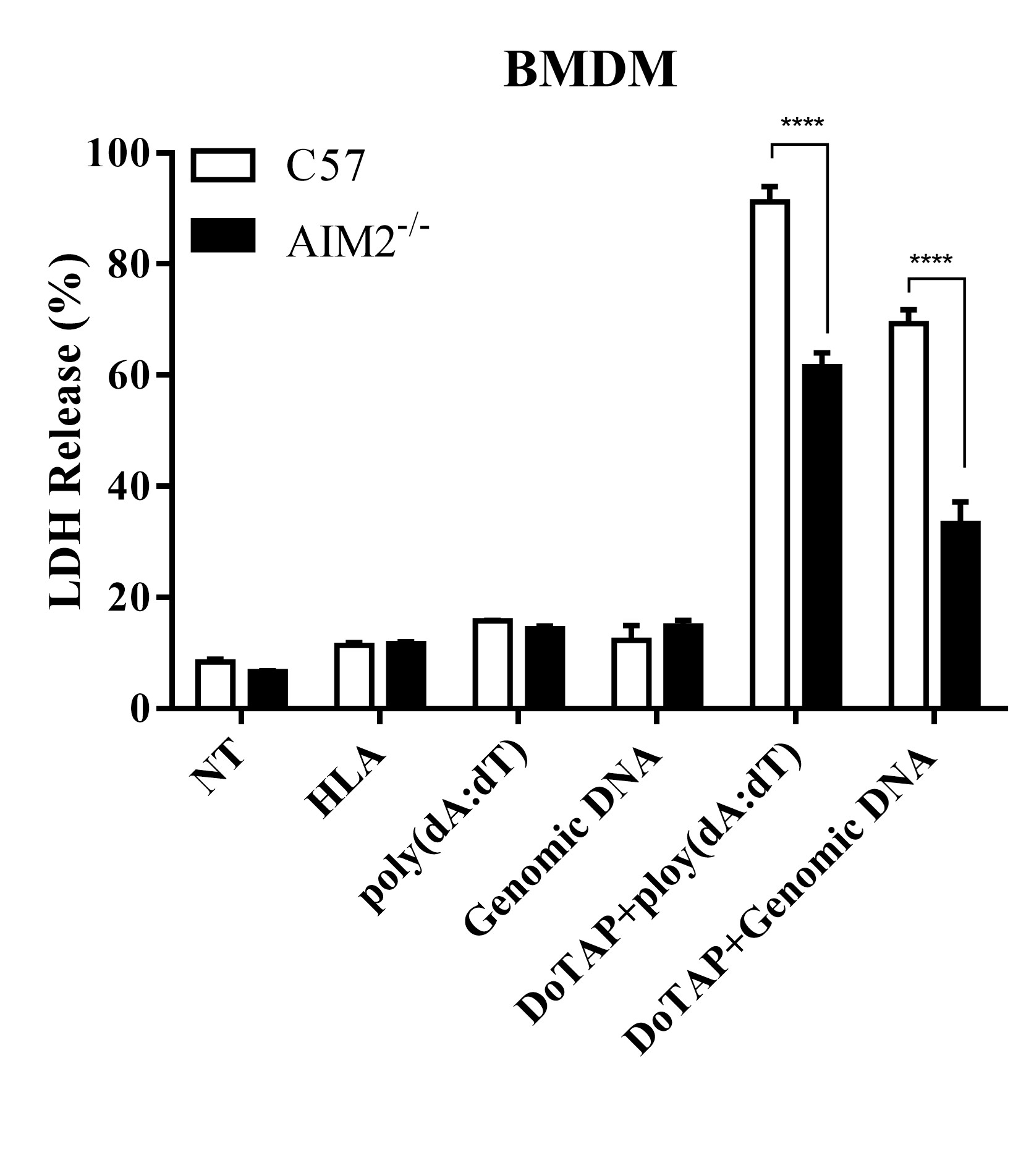

Supplement: Supplemental Material [file KVIR_A_2127209_SM5601.zip › supplementary/Fig S1.jpg]

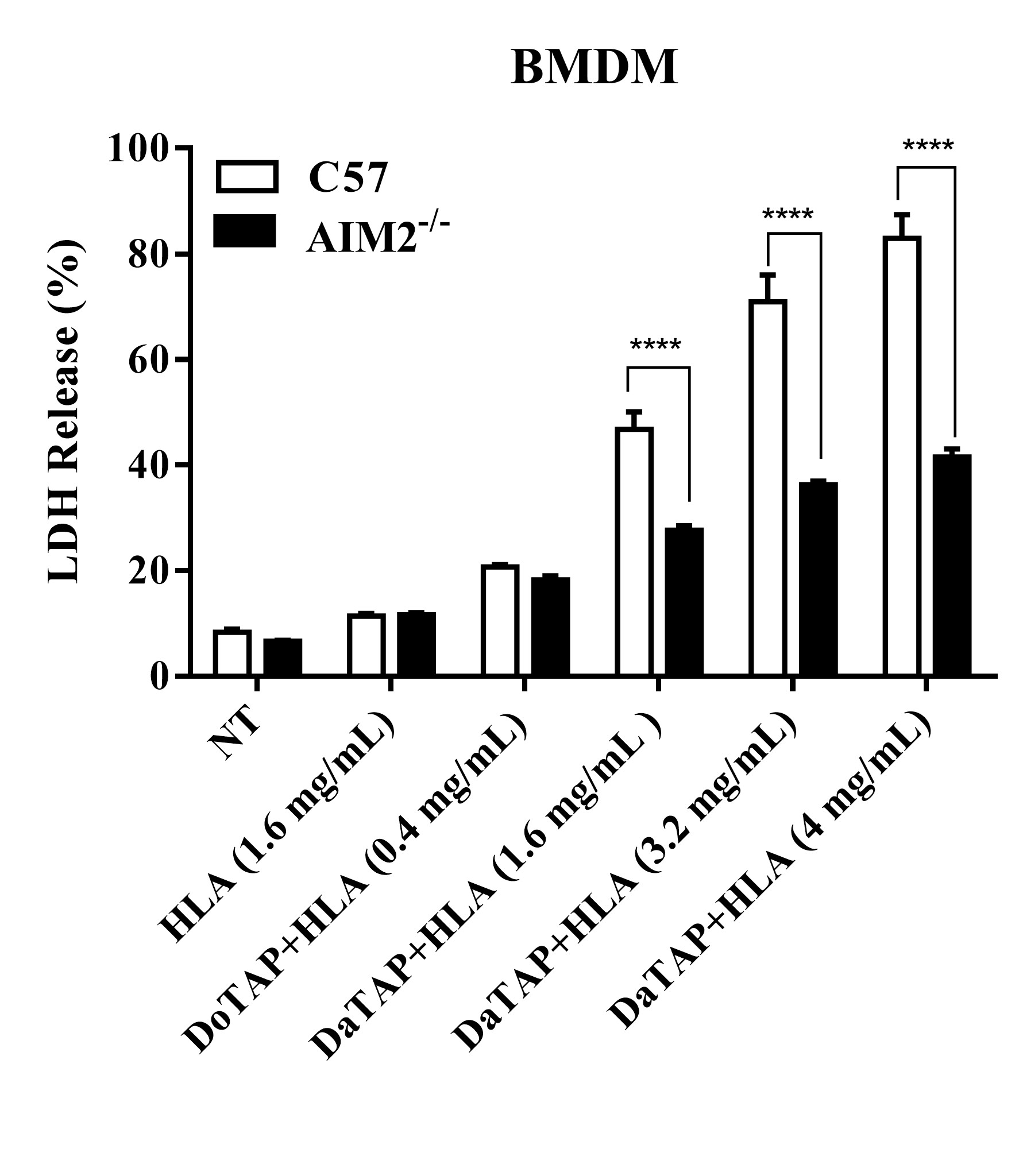

Supplement: Supplemental Material [file KVIR_A_2127209_SM5601.zip › supplementary/Fig S2.jpg]

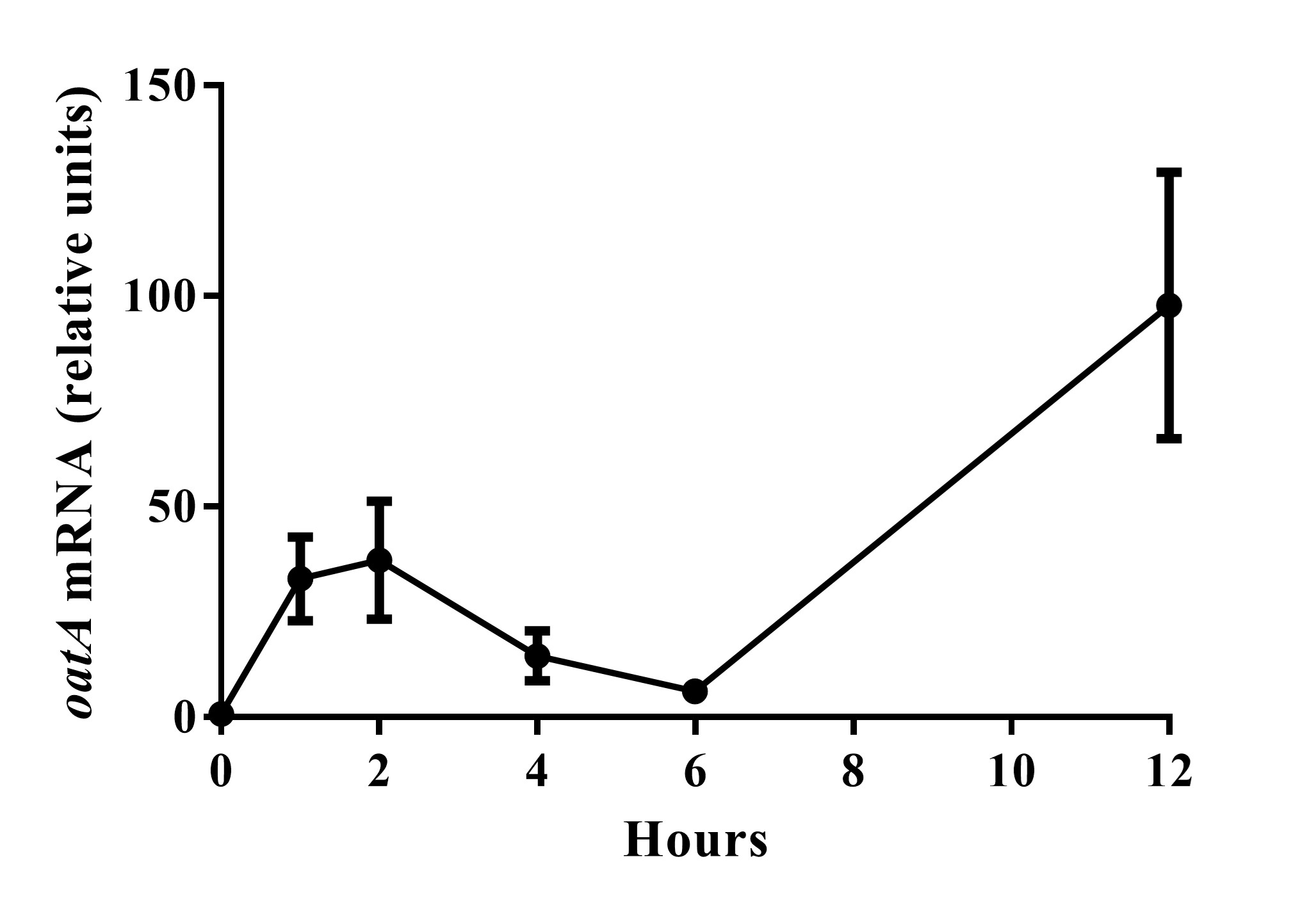

Supplement: Supplemental Material [file KVIR_A_2127209_SM5601.zip › supplementary/Fig S3.jpg]

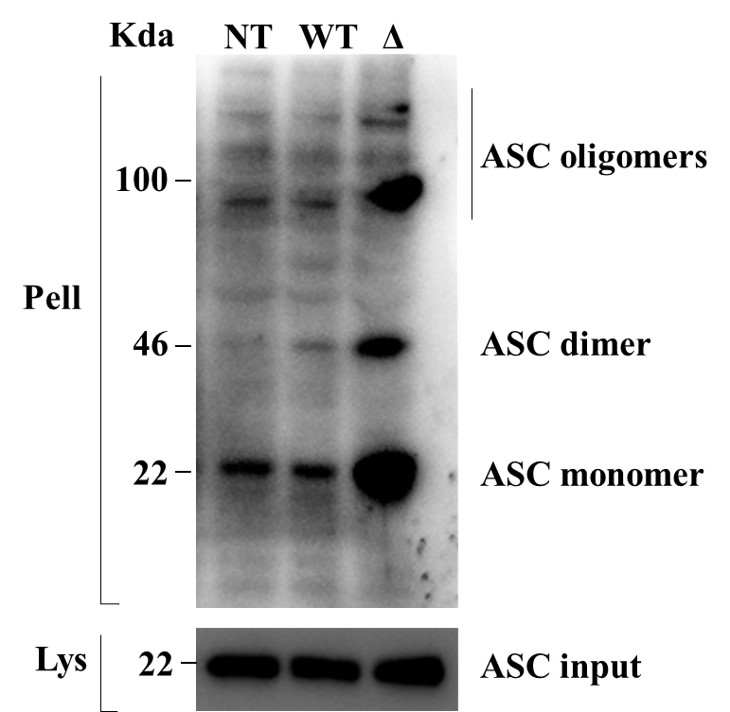

Supplement: Supplemental Material [file KVIR_A_2127209_SM5601.zip › supplementary/Fig S4.jpg]

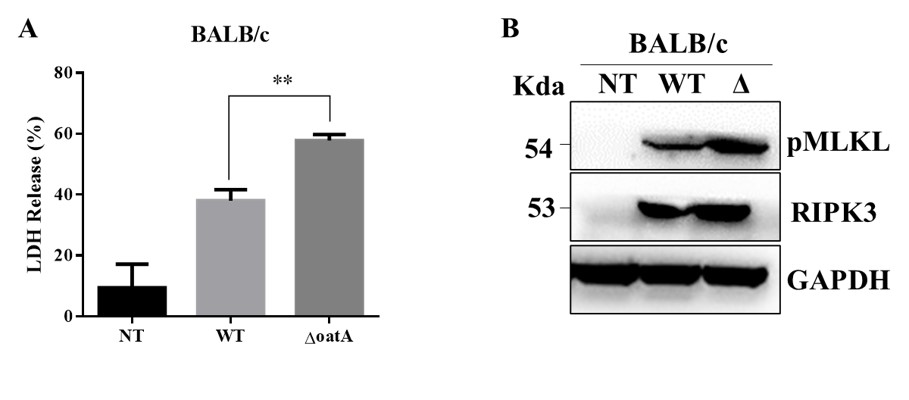

Supplement: Supplemental Material [file KVIR_A_2127209_SM5601.zip › supplementary/Fig S5.jpg]
